# Bioactive Natural Products Targeting Androgen Receptor Signaling in Prostate Cancer: A Systematic Review

**DOI:** 10.3390/cancers18050786

**Published:** 2026-02-28

**Authors:** Febby Pratama, Dhania Novitasari, Richa Mardianingrum, Holis Abdul Holik, Nur Kusaira Khairul Ikram, Muchtaridi Muchtaridi

**Affiliations:** 1 Departement of Pharmaceutical Analysis and Medicinal Chemistry, Faculty of Pharmacy, Universitas Padjadjaran, Jl Ir. Soekarno KM 21, Jatinangor 45363, Indonesia; febby24001@mail.unpad.ac.id (F.P.); dhania@unpad.ac.id (D.N.); holis@unpad.ac.id (H.A.H.); 2 Departement of Pharmacy, Faculty of Health Sciences, Universitas Perjuangan, Jl Peta 177, Tasikmalaya 46115, Indonesia; richamardianingrum@unper.ac.id; 3 Institute of Biological Sciences, Faculty of Science, Universiti Malaya, Kuala Lumpur 50603, Malaysia; nkusaira@um.edu.my; 4 Research Collaboration Centre for Radiopharmaceuticals Theranostic, National Research and Innovation Agency (BRIN), Sumedang 45363, Indonesia

**Keywords:** natural product, phytochemical, androgen receptor, prostate cancer

## Abstract

**Background:** Prostate cancer remains a leading cause of male cancer-related mortality, largely driven by the dysregulated activation of the androgen receptor (AR) signaling pathway. The emergence of resistance, particularly in castration-resistant prostate cancer (CRPC), necessitates the discovery of innovative therapeutic approaches. This systematic review aims to consolidate contemporary evidence regarding natural products as bioactive alternatives capable of targeting the AR signaling axis. **Methods:** Adhering to PRISMA guidelines, a systematic search was conducted across PubMed, Scopus, and ScienceDirect databases. The review identified and qualitatively analyzed 15 original research studies that investigated the efficacy and mechanisms of various natural compounds in modulating AR signaling. **Results:** The analysis reveals that natural products deactivate the AR signaling axis through diverse mechanisms. Neoisoliquiritin and *α*-terthienyl were found to suppress AR expression, activity, and nuclear translocation. Notably, *α*-mangostin facilitates the degradation of the AR-V7 splice variant, a key driver of treatment resistance. Manzamine A inhibits AR biosynthesis by targeting the transcription factor E2F8. Furthermore, alternative pathways are modulated through 5-*α*-reductase inhibition (*Annona muricata* compounds) and the activation of the non-classical membrane receptor ZIP9 by (-)-epicatechin to induce apoptosis. **Conclusions:** The emergence of resistance, particularly in castration-resistant prostate cancer (CRPC), necessitates the exploration of innovative therapeutic approaches. This systematic review consolidates contemporary evidence regarding natural products as potential bioactive alternatives for modulating the androgen receptor (AR) signaling axis. Rather than providing a definitive clinical roadmap, this work establishes a preclinical framework for identifying substances that may deactivate the receptor, break down its resistant forms, or prevent nuclear translocation.

## Simple Summary

Prostate cancer is a leading cause of death in men, often because the disease becomes resistant to standard therapies that target male hormones. This resistance occurs when the cancer’s “control switch”, known as the androgen receptor, continues to drive growth despite treatment. This systematic review examines 15 recent studies to identify natural compounds that can successfully block this switch through various biological methods. We found that these natural substances can deactivate the receptor, break down its resistant forms, and prevent it from reaching the cell’s nucleus. The emergence of resistance, particularly in castration-resistant prostate cancer (CRPC), necessitates the exploration of innovative therapeutic approaches. This systematic review consolidates contemporary evidence regarding natural products as potential bioactive alternatives for modulating the androgen receptor (AR) signaling axis. Rather than providing a definitive clinical roadmap, this work establishes a preclinical framework for identifying substances that may deactivate the receptor, break down its resistant forms, or prevent nuclear translocation.

## 1. Introduction

Prostate cancer is the most common urogenital malignancy in men and the second largest cause of cancer deaths globally. Over 1.4 million prostate cancer cases were reported in 2020, with 375,000 fatalities [1,2,3]. The biochemical factors that cause this disease, including hormonal signals, must be studied to understand why it is so common and devastating. Normal prostate growth and maintenance need androgen signaling (AR) [4]. Testosterone and dihydrotestosterone (DHT) imitate the AR, a ligand-activated transcription factor that regulates prostate cell proliferation, differentiation, and viability genes. The AR signaling system is vital for normal biological development, and its dysregulation contributes to the onset and progression of prostate cancer. Physiologically, negative feedback and post-translational regulation tightly control AR activity. However, when the AR signaling pathway becomes dysregulated, prostate cancer cells continue to grow instead of undergoing apoptosis. To better understand these molecular processes, it is important to examine the structure of the AR. The AR consists of a hinge region, a DNA-binding domain (DBD), an N-terminal domain (NTD), and a ligand-binding domain (LBD) [5]. These domains must remain intact, as alteration can affect receptor function and reduce drug efficacy. Mutations in these regions, especially the LBD, can lead to receptor promiscuity, AR promiscuity in binding to non-androgenic ligands, and resistance to treatment. Medical professionals must utilize standard medical practices, despite their limitations, to tackle these intricate clinical challenges [6,7].

Prostate cancer can be treated using several therapeutic approaches, depending on the stage of the disease, the patient’s overall health, and the risk of metastasis. However, these treatments are often expensive and have severe side effects [8]. Some patients may develop resistance to therapy or even metastasis, making long-term therapy more difficult. These significant deficiencies in the existing standard of treatment underscore the pressing necessity to transition to alternative therapy. Due to the limits of standard medicines, alternative medications are necessary to surmount AR resistance and enhance cancer cell selectivity [9]. Natural products offer a substantial and historically validated repository of selective and effective pharmaceutical alternatives.

Natural products play an essential role in contemporary pharmacology, with more than 60% of FDA-approved anticancer medications derived from or influenced by natural sources [10,11,12,13]. Natural compounds continue to be utilized in cancer therapy due to their unique therapeutic properties, which cannot be completely reproduced by synthetic drugs. Their benefits include a wide range of structures, the ability to optimize biological activity through evolution, multitarget approaches to deal with tumor heterogeneity, and safe profiles produced by biological system co-evolution [14,15]. Nevertheless, challenges related to absorption and delivery require researchers to explore improved strategies for administering these active substances. Approaches such as structural modifications and advanced formulation techniques are essential to preserve biological activity while enhancing the bioavailability of natural compounds [16,17].

Once bioavailability is enhanced, these compounds must also demonstrate the ability to distinguish between healthy and cancerous cells. Phytochemicals have the potential to selectively target cancer cells by exploiting their altered metabolism, membrane composition, and protein expression profiles [18,19,20]. Such interactions can induce cancer-specific cytotoxicity while sparing normal cells. Phytochemicals have the ability to suppress the p53 protein in normal cells, thereby triggering programmed cell death (apoptosis) in tissues with high turnover rates, such as the bone marrow, lymphatic system, hair follicles, and intestinal lining [21,22]. Mechanisms underlying this selectivity include preferential uptake by cancer cells, conversion into toxic compounds inside the cancer cell microenvironment, and the targeting of cancer-specific functions [23]. To effectively translate these strategies into cancer treatments in both laboratory and clinical setting, a robust and well-organized scientific foundation is required. Advancing phytochemicals into viable anticancer therapeutics cancer, demands a focused and diverse research method. Although such strategy is essential, the current literature lacks a comprehensive evaluation of the interactions between these phytochemical-based compounds and androgen receptors, indicating a critical gap in knowledge.

Most existing reviews focus on isolated compounds or broad anticancer processes rather than elucidating how phytochemicals specifically modulate androgen receptor (AR) signaling. This gap is compounded by fragmented and inconsistent findings, resulting in an incomplete understanding of phytochemicals effects on AR pathways. These discrepancies are due to the variations in experimental conditions, including compound purity, methodological approaches, and evaluation criteria, which hinder meaningful comparison across studies. Furthermore, limited application of advanced computational methods and systems biology approaches restricts the ability to model complex interactions between phytochemicals, AR signaling, and cellular networks. Regulatory constraints and stringent approval pathways for natural compounds further impede clinical translation. Considering these gaps and limitation, this review aims to consolidate and clarify current knowledge on phytochemicals as AR antagonists in prostate cancer. This review examines phytochemicals as androgen receptor antagonists in prostate cancer through computational, cellular, and animal studies.

Despite extensive research of androgen receptor biology and increasing interest in phytochemicals as anticancer agents, most existing reviews focus on individual compounds or broad anticancer mechanisms, lacking a systematic, mechanism-oriented synthesis of how natural products modulate androgen receptor (AR) signaling across multiple regulatory levels. Notably, the roles of AR splice variant degradation, upstream transcriptional control, non-canonical membrane androgen receptors, and androgen biosynthesis inhibition remain fragmented and inconsistently discussed in the literature. To address this knowledge gap, the present systematic review critically integrates computational, in vitro, and in vivo evidence on natural-product-derived compounds targeting AR signaling in prostate cancer. By categorizing these compounds based on their molecular mechanisms of action, this review aims to elucidate emerging therapeutic paradigms and identify promising lead structures for overcoming resistance in advanced and castration-resistant prostate cancer.

## 2. Search Strategy

This systematic review was conducted in accordance with PRISMA guidelines. The review protocol was registered on the Open Science Framework (OSF) with the registration number 10.17605/OSF.IO/XBST8. A comprehensive literature search was performed using PubMed, Scopus, and ScienceDirect to identify relevant studies published between 2016 and 2025. The search method used keywords and Boolean operators: ((“prostate cancer” OR “prostatic neoplasms”)) AND ((“androgen receptor” OR “AR”)) AND ((“natural product” OR “phytochemical” OR “bioactive compound” OR “herbal medicine”)). While the initial Boolean-based search strategy yielded a substantial volume of 6328 records, a rigorous multistage screening process was applied to ensure that the final qualitative synthesis provided a focused mechanistic roadmap. During the screening of 5911 unique records, 5896 were excluded primarily because they focused on broad anticancer phenotypes or isolated antioxidant properties without investigating specific molecular interactions with the androgen receptor (AR) axis.

A multistage screening process was used to identify eligible studies. All retrieved records were first compiled, and duplicate entries were removed. Titles and abstracts of the remaining articles were then screened to assess relevance. Studies were included for full-text evaluation if they were original research articles investigating the effects of natural products or their derivatives on androgen receptor signaling in prostate cancer models. Review papers, conference abstracts, and studies unrelated to the topic were excluded from further consideration.

The final articles data was extracted independently. The first author and publication year, the natural product or bioactive compound investigated, the experimental model (in vitro cell lines, in vivo animal models, in silico computational studies), the primary androgen receptor mechanism of action, and key outcomes were extracted. The final qualitative synthesis included all 15 eligible studies, and their findings were analyzed to determine how natural bioactive compounds interact with AR through various mechanisms, including the suppression of AR expression, activity, and nuclear translocation (neoisoliquiritin and *α*-terthienyl), degradation of the AR-V7 splice variant (*α*-mangostin), inhibition of AR biosynthesis by novel targets such as the transcription factor E2F8 (manzamine A), and modulation of alternative pathways, such as 5-*α*-reductase inhibition (*Annona muricata* compounds) or activation of the non-classical membrane receptor ZIP9 to induce apoptosis ((-)-epicatechin) in prostate cancer models within in silico, in vitro and in vivo studies. The inclusion of only 15 studies was a deliberate decision to maintain scientific stringency. We prioritized original research that moved beyond general cytotoxicity to elucidate precise regulatory levels, such as AR splice variant (AR-V7) degradation, upstream transcriptional control (E2F8), and non-classical membrane receptor (ZIP9) activation. Studies utilizing poorly characterized herbal extracts or those lacking defined molecular targets within the AR signaling cascade were excluded to prevent the reporting of ambiguous, off-target biological effects.

## 3. Data Extractions

**Figure 1:**
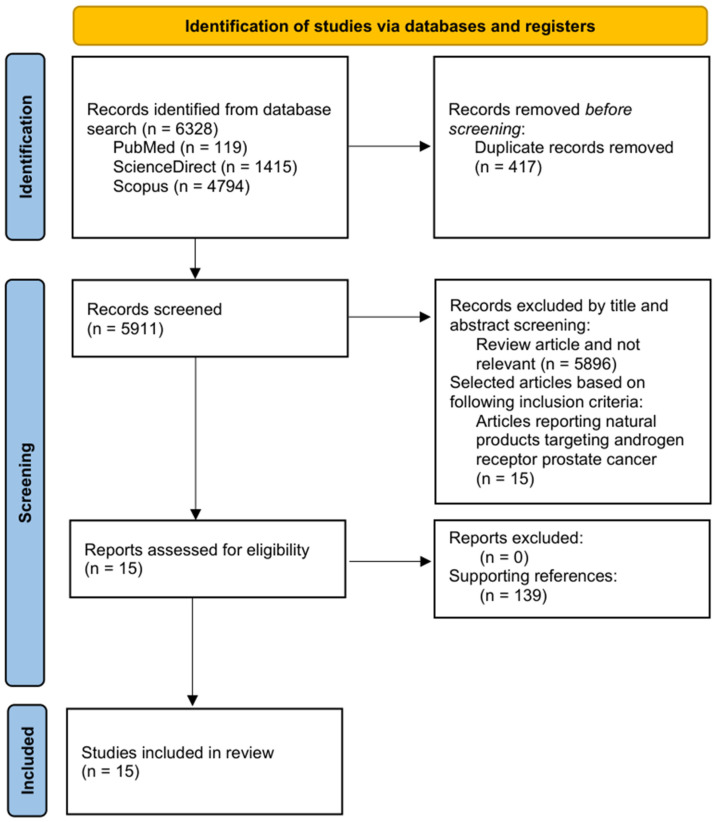
A flow diagram of the search strategy according to PRISMA guidelines.

The initial database search yielded 6328 results: 119 from PubMed, 1415 from ScienceDirect, and 4794 from Scopus. To ensure the dataset contained only unique and relevant publication, duplicates and non-relevant studies were removed. After eliminating 417 duplicates, 5911 articles remained for title and abstract screening. During this stage, 5896 publications were excluded because they were reviews articles or were not related to the scope of this review. Only 15 publications met the inclusion criteria, focusing specifically on natural compounds that modulate androgen receptor signaling in prostate cancer. These 15 studies were then subjected to full-text evaluation, and no additional articles were excluded during eligibility assessment (n = 0). The final qualitative synthesis included all 15 eligible studies, and their findings were analyzed to determine how natural bioactive compounds interact with AR through various mechanisms, including the suppression of AR expression, activity, and nuclear translocation (neoisoliquiritin and *α*-terthienyl), degradation of the AR-V7 splice variant (*α*-mangostin), inhibition of AR biosynthesis by novel targets such as the transcription factor E2F8 (manzamine A), and modulation of alternative pathways, such as 5-*α*-reductase inhibition (*Annona muricata* compounds) or activation of the non-classical membrane receptor ZIP9 to induce apoptosis ((-)-epicatechin) in prostate cancer models within in silico, in vitro and in vivo studies. Figure 1 illustrates the comprehensive study selection process in accordance with the PRISMA flow diagrams. Additionally, the review was supported by the utilization of 138 references, thereby providing a robust scientific foundation.

This systematic review examines natural compounds that inhibit the activation of AR activation in prostate cancer through different molecular pathways. Polyphenolic compounds, which are one of the main inhibitors, were evaluated first. Several polyphenols, including neoisoliquiritin, alpinumisoflavone, hispidin, baicalein, and tangeretin, demonstrate AR-suppressive activity. This is because research and review publications extensively discuss this class of compounds for their inhibitory effects on AR. Polyphenols show anticancer effects through various mechanisms, such as modifying signaling pathways to eliminate cancer cells, inhibiting cell cycle events, and inducing apoptosis. Additionally, they regulate the activities of enzymes involved in tumor cell proliferation [24,25]. Neoisoliquiritin inhibits AR expression, nuclear translocation, and transcription activity, leading to cell-cycle arrest and reduced tumor proliferation [26]. Alpinumisoflavone also affect cancer cell metabolism by lowering AR and PSA expression and disrupts lipid metabolism by inhibiting FASN and HMGCR, resulting in apoptosis and metabolic stress [27]. Hispidin exhibits both antiproliferative and antimetastatic effects by suppressing AR signaling and downregulating metastatic enzymes such as MMP 2 and MMP 9, while promoting PARP and caspase 3 cleavage [28]. Baicalein downregulates HMMR to decrease AR transcription and motility signaling [29], while tangeretin targets AR-AKT pathways and restores Connexin 26, sensitizing cells to chemotherapy [30]. Recent research on catechins, instead of flavonoids, uses biological testing and computer modeling to show how AR interacts with natural chemicals [31].

While computational studies reveal substantial binding affinity to the AR-ligand-binding domain (LBD), tea-catechin-based drugs have AR-independent lethal effects, suggesting competitive inhibition with endogenous androgens. A computational method has identified strong AR inhibitors from grape stilbenoids and other plant sources. In silico modeling shows strong AR binding affinity and stability for grapevine stilbenoids like cis-piceid and procyanidins. Alkaloids suppress AR synthesis differently to polyphenols, which target receptor proteins. In contrast to other alkaloids, manzamine-A inhibits the transcription factor E2F8, preventing AR-FL and AR-V7 production. Other lotus seed alkaloids impair survival signaling pathways that support AR function and transcription factors. Lotus alkaloids (neferine, liensinine, isoliensinine) indirectly decrease AR expression by inactivating PI3K/AKT. Triterpenoids and thiophenes display indirect AR inhibition through gene expression suppression and other protective mechanisms. Similarly, *α*-terthienyl reduces AR expression and prevents nuclear translocation, while lupeol boosts antioxidant capacity, suppresses AR mRNA, inhibits AKT signaling, and activates P27. Despite inhibiting expression and translocation, physically eliminating the receptor is a more aggressive technique, especially for resistant splicing variants. In castration-resistant prostate cancer (CRPC), *α*-mangostin overcomes treatment resistance by promoting AR-FL and AR-V7 degradation via BiP-mediated ubiquitination. In addition to directly damaging or blocking the receptor, cutting off energy sources that promote the androgen signaling pathway works. Natural compounds suppress AR signaling directly and via auxiliary mechanisms. Plant compounds can suppress the enzyme that produces the greatest androgen, a useful new target. Compounds from *Annona muricata* and *Nelumbo nucifera* block 5-*α*-reductase, inhibiting the synthesis of DHT, a powerful AR ligand. Lotus seed alkaloids also suppress cancer growth signals enzymatically. Neferine-type alkaloids block 5-*α*-reductase, lowering testosterone to DHT conversion. Some therapeutic natural compounds activate cell surface non-classical androgen receptors instead of suppressing signals.

Recent therapies use non-classical processes. Epicatechin, a ZIP9 membrane-associated AR agonist, switches survival signals into proapoptotic pathways in AR-negative PC-3 cells. Epicatechin modulates androgen signaling, while various additional substances modulate the hormone receptor system to stop cell development. Additionally, the furanoflavonoid karanjin activates ERβ and inhibits AR, causing ROS-mediated apoptosis and G2/M arrest, indicating significant hormonal and cytotoxic regulatory potential. When taken together, these data show that natural compounds help combat prostate cancer. These natural chemicals, which combine non-genomic regulation, enzyme inhibition, AR suppression, and degradation, offer promising treatments for therapy-resistant prostate cancer.

## 4. Discussion

### 4.1. Natural Products Mechanism in Action Targeting Androgen Receptor Prostate Cancer

Plants produce phytochemicals as a defense against predators and pathogens and in reaction to environmental stress [32]. Major classes include polyphenols (stilbenes, phenolic acids, flavonoids), terpenoids (sesquiterpenes, monoterpenes, triterpenes), alkaloids, and sulfur-containing chemicals [33]. These chemical configurations allow phytochemicals to interact with cancer cells in numerous ways, providing many therapeutic effects. The antitumor mechanisms of phytochemicals include mitochondrial pathway apoptosis, cell cycle arrest at critical points, inhibition of tumor neovascularization and metastasis, inhibition of inflammation cascades, induction of detoxification enzymes, and epigenetic modifications [34,35,36]. Cancer is usually caused by complex, interconnected cellular regulatory mistakes; therefore, this wide mechanistic capability is very useful [37]. Phytochemicals’ multitarget action helps overcome tumor heterogeneity and cancer cell resistance [38]. To understand how these multitarget drugs treat prostate cancer, one must first understand the androgen receptor signaling pathway.

**Figure 2:**
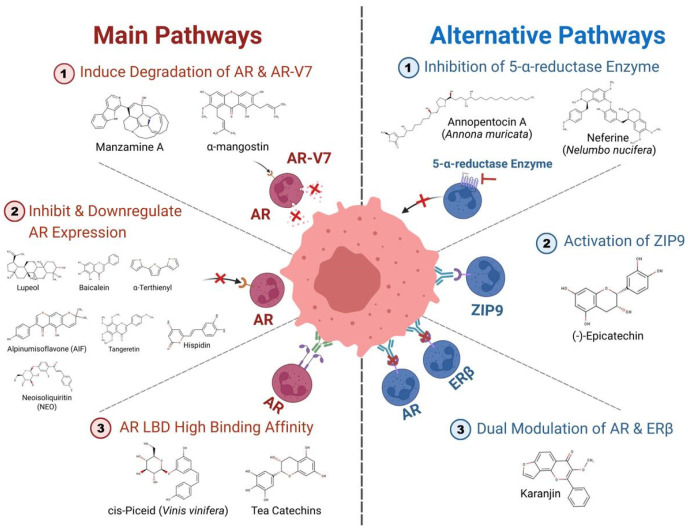
Work of bioactive compounds from natural products on androgen receptor pathways in prostate cancer (created with BioRender.com).

In prostate cancer, androgen binding, nuclear translocation, receptor dimerization, and binding of the androgen receptor complex to androgen response elements (AREs) in the promoter sites of target genes like PSA, TMPRSS2, and KLK3 comprise the AR signaling pathway [39,40,41]. Genetic alterations in this signaling system cause cancer to progress to more dangerous stages. Amplification of the AR gene, production of splice variants such AR-V7, and overexpression of coactivator proteins are key steps in prostate cancer progression to advanced stages as we can see in Figure 2 [42,43]. Due to the disease’s dependence on this pathway, medical science has spent decades developing standard treatments. Huggins and Hodges pioneered androgen deprivation therapy (ADT) for advanced prostate cancer, which has been routine treatment for almost 80 years [44].

This treatment has evolved from basic interventions to specialized medical and surgical methods to lower hormone levels. ADT usually involves surgical or chemical castration combined with antiandrogen therapy such as bicalutamide, flutamide, or nilutamide [45]. Knowledge in receptor biology has facilitated the development of more effective, next-generation pharmaceutical drugs. For example, enzalutamide and apalutamide exhibit higher receptor affinity and inhibit AR activity by blocking nuclear translocation and transcriptional activation of AR target genes [46,47,48]. In addition, advanced therapeutic strategies have been established to block activating hormone production or supply while simultaneously employing receptor-direct antagonism.

Abiraterone acetate inhibits CYP17A1, a major regulator of androgen steroid production. Traditional androgen deprivation therapy prolongs survival but is restricted [49,50,51,52]. When these medicines fail, the tumor adjusts to survive and grow in a hormone-depleted environment, changing the prognosis. Most individuals develop castration-resistant prostate cancer (CRPC) after 18–24 months, which reactivates AR signaling despite low testosterone levels [53]. Disease returns in low-androgen conditions due to the tumor’s complex molecular alterations to evade treatment. Amplification, gain-of-function mutation, AR splice variant expression, intracrine androgen production, and alternate survival pathways are resistance mechanisms [54,55,56]. Tumor resistance and physically hard treatments degrade the patient’s daily life. Long-term ADT causes devastating side effects like hot flashes, osteoporosis, sarcopenia, and metabolic syndrome, highlighting the need for more targeted and acceptable treatments [57,58]. Advanced discovery tools capable of predicting chemical efficacy in advance of physical testing are critical to overcoming resistance and toxicity.

Computational drug discovery reveals binding processes, identifies pharmacophores, and predicts ADMET properties before experimental validation [59]. Recent improvements have improved computational models. Artificial intelligence and machine learning have improved computer model efficacy, accuracy, and predictive capacity in drug discovery processes [60]. Computational methods find drug candidates, which biological labs must verify. In vitro cell culture methods provide cytotoxicity detection, mechanism analysis, and dose–response relationships. To connect simple cell cultures with complex human biology, researchers are employing more complex culture models. Complex cell culture methods like 3D organoids and co-culture systems imitate in vivo circumstances better [61]. A compound’s systemic safety and efficacy must be tested in humans, even with enhanced cellular models. Before human trials, in vivo animal models demonstrate efficacy and safety. This study chose complementary methodologies from recent research. Preclinical research using omics technologies (genomics, proteomics, metabolomics) can also analyze chemical mechanisms of action and identify biomarkers [62].

These featured studies used many complimentary methodologies to understand the substances mechanisms of action. Reliable biological and computational methods were used in this research. Most research used androgen-sensitive (LNCaP), castration-resistant (CRPC) (C4-2, 22Rv1, VCaP), and androgen-independent (PC-3, DU-145) prostate cancer cell lines. To anticipate the binding affinity of bioactive compounds to target proteins like the androgen receptor (AR), molecular docking was widely used, employing PDB structures like 5T8E and 2Q7I, as well as 5-*α*-reductase and ZIP9. Several significant research works validated in vivo treatment efficacies using mouse xenograft models. These in vivo trials indicated that natural substances could inhibit tumor growth. Studies on manzamine-A, *α*-terthienyl, neoisoliquiritin, and *α*-mangostin showed significant tumor growth inhibition, directly affecting the androgen receptor suppresses tumors.

### 4.2. Downregulation of AR Expression, Activity, and Translocation

#### 4.2.1. Compounds Inhibiting AR Transcriptional Activity and Translocation

**Figure 3:**
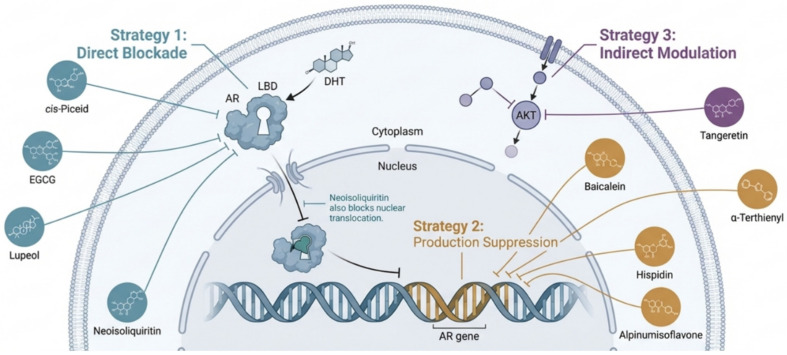
Schematic representation of the multitargeted inhibition of androgen receptor (AR) signaling by bioactive natural products (created with BioRender.com).

Most bioactive substances directly inhibit AR function to fight cancer [63]. This method suppresses AR synthesis, activation, and nucleus transcription. Flavonoids, catechins, and polyketides reliably reduce AR [64]. This direct inhibition is seen in licorice root flavonoid neoisoliquiritin. Chen et al. studied licorice-derived chalcone flavonoid neoisoliquiritin (NEO) [26]. This study found NEO to be a highly selective androgen receptor (AR) signaling inhibitor. NEO strongly inhibited AR-dependent LNCaP prostate cancer cell proliferation while having low cytotoxicity in AR-independent PC3 cells, indicating an AR-centric mechanism of action [65]. The study showed that NEO knocks down the AR system by downregulating AR production, inhibiting nuclear translocation of residual receptor protein, and suppressing AR transcriptional activity. This thorough inhibition arrested the G0/G1 cell cycle, proving its antiproliferative properties [66]. After NEO treatment, RNA-seq showed that AR signaling was the most affected pathway. NEO showed considerable tumor suppression in a mouse xenograft model, demonstrating its therapeutic potential as a natural AR-targeting drug [26]. While neoisoliquiritin directly hits the receptor, flavonoids such as alpinumisoflavone disturb cell metabolism further as we can see in Figure 3.

Basavaraj et al. [27] found that the isoflavonoid compound alpinumisoflavone (AIF) modulates AR signaling and metabolic pathways necessary for prostate cancer cell survival. AIF dramatically lowers AR and PSA mRNA and protein expression in androgen-sensitive LNCaP and C4-2 cells [67]. Antiandrogen action on LNCaP and LAPC4 cells resulted in a decrease in androgen receptor (AR) protein levels [67]. In contrast, a study was conducted on C4-2 cells to investigate the reduction in a marginal AR protein following treatment [68,69]. Direct AR suppression lowered cell viability, invasion, and migration and induced apoptosis via PARP and caspase-3 breakage [70]. In addition, AIF targeted FASN and HMG-CoA Reductase, which are involved in cholesterol and lipid biosynthesis [27]. This twofold disturbance affected cancer cell energy balance, membrane stability and integrity, and steroid precursor production due to AR signaling and lipid metabolism’s strong relationship. AIF is a strong antiproliferative drug throughout disease stages because it inhibits AR-driven transcriptional programs and metabolic networks that preserve tumor survival [71]. Wang et al. reported that AIF possesses a significant mechanism as an anticancer agent, capable of suppressing tumor growth and metastasis. This mechanism involves the modulation of the Akt/miR-101/RLIP76 signaling axis [72]. Other polyphenols also inhibit multitargeted cancer, but each has its own mechanism [73].

#### 4.2.2. Compounds Suppressing AR Protein and Gene Expression

Hispidin, baicalein, and tangeretin are other polyphenols that downregulate androgen receptors (ARs) but have different mechanisms. Chan et al. found that hispidin, a phenolic polyketide, targets the AR and metastatic pathways in prostate cancer [74]. Hispidin reduces AR protein levels in androgen-sensitive (LNCaP) and castration-resistant (C4-2) cells, suggesting that it could treat early-stage and therapy-refractory illness. Suppressing the AR signaling pathway significantly reduces metastatic potency. Hispidin inhibits the activity of AR and metastatic enzymes such as MMP 2 and MMP 9, while simultaneously inducing PARP and caspase 3 cleavage [74]. The expression levels of MMP-2 and MMP-9 can serve as predictive indicators of prostate tumor behavior following prostatectomy, irrespective of whether surgical margins are positive or negative [75]. Prostate regrowth appears to involve a more effective participation of MMP-2 in both the epithelial and stromal compartments, while MMP-9 plays a significant role in late prostate atrophy and early regrowth [76]. Therefore, the inhibition of MMP2 and MMP9 results in the suppression of metastasis in prostate cancer. Fewer MMP-2 and MMP-9, important proteases in extracellular matrix destruction and cancer invasion, were expressed and active [77]. MMP2 and MMP9 also play a crucial role in the inflammatory process in numerous cancer cases. These enzymes are essential for various physiological functions, including tissue remodeling, inflammation, and wound healing [78,79,80]. Wound healing and transwell migration showed prostate cancer cell motility reduction [81,82]. The other mechanism shows that hispidin promotes apoptosis through caspase-3 and PARP cleavage, validating its multifunctional anticancer potential. Wang et al. reported that hispidin modulated the MAPK and NF-kB signaling pathways, leading to a downregulation of AKT phosphorylation and inhibition of ferroptosis signaling pathways. Interestingly, scavenging ROS or inhibiting these signaling pathways reversed hispidin-induced apoptosis, ROS levels, mitochondrial dysfunction, and ferroptosis [83,84]. These findings suggest that hispidin may hold promise as a potential prostate cancer treatment. While hispidin targets metastasis, flavones like baicalein affect gene transcription early on.

Baicalein, a *Scutellaria baicalensis* flavone, shows intriguing dual-mechanism pharmacology [29]. Baicalein specifically inhibits AR promoter transcription in LNCaP prostate cancer cells. Luciferase-based cell reporting assays directly limit AR mRNA production, reducing AR protein levels and PSA target gene expression [85]. Baicalein’s molecular antiandrogenic action is confirmed. Baicalein also inhibits the HMMR/RHAMM oncogene, which is overexpressed in poor-prognosis tumors, in addition to androgens. Baicalein treatments dramatically inhibit HMMR expression, reducing LNCaP cell growth and migration [86]. Baicalein may be a multitarget treatment for AR-dependent prostate cancer due to its AR suppression and HMMR inhibition of proliferation and motility [87]. Flavonoids like tangeretin can restore tumor defenses in drug-resistant situations.

Polymethoxylated flavone tangeretin inhibits AR and AKT kinase, disrupting linked carcinogenic pathways in CRPC cells (C4-2 cell line) [30]. Tangeretin also upregulated Connexin 26 (Cx26), a gap junction protein downregulated in prostate cancer, restoring intercellular communication and suppressing tumors [88]. Knockdown tests showed that Cx26 deletion partially restored cells from tangeretin-induced growth inhibition, supporting its functional importance [89]. Tangeretin also increased the cytotoxic effects of cisplatin and sorafenib, suggesting its use as an adjuvant to overcome chemoresistance in advanced prostate cancer [90]. Besides these promising flavonoids, tea has strong chemicals that are being re-evaluated using current methods [91]. Table 1 show us the summary of bioactive compounds targeting androgen receptor signaling in prostate cancer.

Muriuki et al. used in vitro and in silico methods to assess the antiprostate cancer capabilities of various tea catechins [97]. The in vitro studies used the androgen receptor (AR)-negative DU-145 cell line, while the computational component revealed molecular evidence of an AR-dependent biochemical process. Purple tea extract had the strongest dose-dependent antiproliferative effects in in vitro experiments. This suggests that tea polyphenols may be harmful without the normal AR signaling mechanism. In addition, the in silico study examined key catechin-AR interactions. Catechins, especially EGCG, interact significantly in the ligand-binding domain (LBD) of the AR protein (PDB ID: 5T8E) [101] according to molecular docking modeling [102]. This high binding affinity shows that there is a competitive inhibitory mechanism with natural androgens like testosterone and DHT. These drugs may obstruct the LBD, inhibiting androgen binding and receptor activation, hence diminishing downstream signaling in AR-positive cells [103]. This study demonstrates the dual mechanistic potential of tea extracts: an AR-independent antiproliferative effect in vitro and direct AR inhibition in silico. These results indicate that AR-positive prostate cancer models warrant investigation. Computers have demonstrated that grapevine compounds inhibit the androgen receptor similarly to tea.

Olubode et al.’s in silico study corroborates the efficacy of *Vitis vinifera* (grape) phytochemicals as direct androgen receptor (AR) inhibitors [95]. This in silico study used an intricate molecular modeling approach to identify natural compounds that interact with the AR LBD similarly to commercially employed antiandrogens such as bicalutamide. Molecular docking demonstrated that five primary compounds cis-piceid, cis-astringin, gallocatechin, procyanidin B3, and procyanidin C1 exhibited lower binding energies than bicalutamide, signifying enhanced and more stable binding affinity. Additional molecular interaction studies revealed that hydrogen bonding and pi-stacking interactions involving the AR active site amino acid residues LEU 704, GLN 711, and TRP 741 contributed to the strong binding affinity observed. Molecular dynamics simulation further supported the stability of these interactions, particularly for cis-piceid, which emerged as the most promising candidate. ADMET prediction indicated favorable pharmacokinetics properties and low toxicity. Collectively, these findings provide strong computational evidence that *Vitis vinifera*-derived stilbenoids and procyanidins represent viable scaffolds for the development of novel direct AR antagonists. Interestingly, while polyphenols predominantly exert their effects by directly targeting the receptor protein, alkaloids tend to suppress receptor production, suggesting complementary mechanistic roles across compound classes.

Liu et al. reported that bisbenzylisoquinoline alkaloids neferine, liensinine, and isoliensinine from lotus seeds suppress AR protein expression by inactivating the PI3K/AKT signaling pathway [94]. Through phosphorylation, strengthening, and activation of the AR transcription activity, the PI3K/AKT pathway is the major cancer cell survival route [104]. Alkaloids indirectly affect AR protein expression and instability by blocking this major route. Recent research shows that natural chemicals can disrupt oncogenic connections that enable AR activity, causing apoptosis and autophagy induction and limiting prostate cancer cell growth and migration. Besides alkaloids, triterpenes such as lupeol can block the receptor and protect the cell from oxidative damage.

Nezami Majd et al. (2024) investigated the effects of lupeol in hormone-receptor-positive cancer cells. [98]. Lupeol significantly reduced AR gene expression at the mRNA level in LNCaP prostate cancer cells, suggesting transcriptional intervention comparable to baicalein or AR mRNA destruction before translation. This inhibition decreased LNCaP cell viability and cytotoxicity dose-dependently [105].

The study further demonstrated that lupeol enhances antioxidant capacity in prostate cancer cells, suggesting that its anticancer effect arise not only from inhibiting the oncogenic AR pathway but also from restoring redox equilibrium. These findings highlight lupeol as a multifunctional natural compound with both antiandrogenic and cytoprotective activities. Additionally, marigold-derived *α*-terthienyl exerts antiandrogenic effects by physically preventing the androgen receptor from entering the cell nucleus, further illustrating the mechanistic diversity of natural AR-modulating agents.

Gan et al. (2022) found that *α*-terthienyl, an oligothiophene derivative from marigold flowers, has anticancer properties, supported by complex chemical mechanism and preclinical validation [93]. This study shows that these drugs have considerable anticancer action, primarily through AR signaling pathway blockage. AR protein and mRNA suppression and receptor nuclear translocation inhibition were found in in vitro studies. In hormone-resistant 22Rv1 cells and androgen-sensitive LNCaP cells, both mechanisms of treatment completely stopped androgen signaling. Molecular suppression of *α*-terthienyl led to considerable reduction in animal xenograft models, indicating therapeutic promise without cellular damage. This research also ties lower AR expression to functional effects, including synergistic PI3K/AKT prosurvival pathway suppression and a large rise in P27 expression. By reactivating the cell cycle regulatory mechanism, *α*-terthienyl may be an effective prostate cancer treatment. While preventing AR function is effective, more severe cases, particularly those involving drug-resistant AR variants, may necessitate strategies that directly degrade or eliminate aberrant receptor forms.

#### 4.2.3. Structural Considerations and Structure-Activity Relationships (SAR)

The potency and specificity of natural products in modulating the androgen receptor (AR) signaling axis are fundamentally rooted in their diverse chemical scaffolds. A critical observation emerging from this systematic review is the correlation between specific structural motifs and the resulting mechanistic interference with the AR pathway. The majority of compounds identified as direct inhibitors of AR function, specifically those that downregulate expression and activity through direct binding, share a polyphenolic or flavonoid core. For instance, neoisoliquiritin (a chalcone), alpinumisoflavone (an isoflavone), baicalein (a flavone), and tangeretin (a polymethoxylated flavone) all utilize a variant of the chromane scaffold. These compounds typically possess a high degree of hydroxylation or methoxylation, which facilitates stable interactions within the AR ligand-binding domain (LBD). Computational modeling across several studies indicates that these phenolic rings engage in pi-stacking and hydrogen bonding with critical residues in the AR active site, most notably LEU 704, GLN 711, and TRP 741. These interactions mimic the binding posture of endogenous androgens like testosterone and dihydrotestosterone (DHT), thereby enabling competitive inhibition.

In contrast, compounds that exert their effects through indirect modulation or the inhibition of AR synthesis often possess significantly different structural architectures. The bisbenzylisoquinoline alkaloids derived from lotus seeds, such as neferine, liensinine, and isoliensinine, exhibit large, nitrogen-containing heterocyclic rings. Their mechanism does not rely on direct LBD binding but rather on the inactivation of the PI3K/AKT survival pathway, which indirectly reduces AR protein stability and transcriptional activity. Similarly, the marine-derived alkaloid manzamine-A possesses a complex polycyclic system that specifically disrupts the interaction between the transcription factor E2F8 and the AR gene promoter, rather than the receptor protein itself.

Furthermore, the triterpenoid class, represented by lupeol, exhibits a pentacyclic steroid-like structure. This structural similarity to steroid hormones likely contributes to its ability to modulate AR gene expression at the mRNA level and restore redox equilibrium within the cellular environment. Finally, specialized structures like the xanthone core of α-mangostin enable unique “seek and destroy” tactics. Its distinct planar ring system facilitates selective interaction with the BiP (Binding immunoglobulin protein) chaperone, triggering the proteasomal degradation of both full-length AR and the therapy-resistant AR-V7 variant. Collectively, these findings suggest that while polyphenols are optimized for direct receptor blockade, alkaloids and terpenoids offer diverse alternative scaffolds for targeting the wider AR regulatory network.

### 4.3. Androgen Receptor Degradation Enhancement (Including Splice Variants)

Variants of AR, notably AR-V7, pose a significant challenge in treating castration-resistant prostate cancer (CRPC) [106]. This variation remains active despite androgenic ligands. Because this variant lacks the ligand-binding domain (LBD), the primary target of hormonal agents such as enzalutamide and abiraterone target, it plays a major role in therapeutic resistance [107]. Notably, several natural products have been shown to selectively induce AR-V7 degradation via proteasome-mediated pathways, presenting promising therapeutic strategies [108]. For example, studies on mangostin-derived compounds identified molecular mechanisms capable of destabilizing and degrading the androgen receptor, offering a potential breakthrough in targeting drug-resistant variants [92].

**Figure 4:**
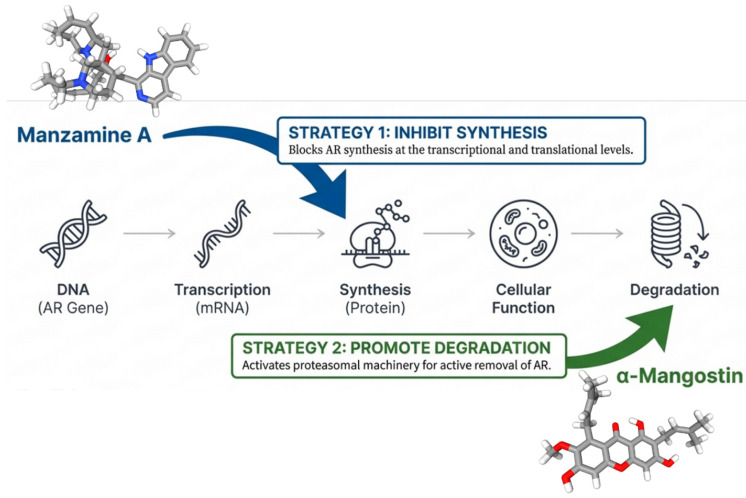
Distinct mechanisms of AR protein depletion by manzamine-A and *α*-mangostin (created with BioRender.com).

As we can see in Figure 4, alkaloids exhibit diverse mechanisms. For example, manzamine-A, a marine β-carboline alkaloid, has been shown to inhibit AR synthesis rather than directly interacting with the receptor protein. Karan et al. reported that the transcription factor E2F8 is a novel and essential repressor of AR gene transcription, creating a new prostate cancer therapeutic vulnerability, where manzamine-A blocks the E2F8 AR gene promoter connection, stopping transcriptional AR production [96]. To prevent resistance, the method stops full-length AR (AR-FL) and the therapy-resistant variant AR-V7 from forming [109]. In in vivo animals, systemic manzamine-A significantly inhibited prostate tumor growth, demonstrating its translational potential [110]. While preventing the receptor from being produced is a powerful strategy, other natural compounds take a more aggressive approach by physically dismantling the receptor protein itself.

In 2023, Nauman et al. explored the therapeutic potential of *α*-mangostin, a xanthone derived from the pericarps of mangostin, making significant contributions to the comprehension of its anticancer mechanisms [92]. *α*-mangostin shows that natural compounds can effectively combat AR using “seek and destroy” tactics. *α*-mangostin effectively degraded both full-length AR (AR-FL) and the therapeutically relevant AR-V7 variant. LNCaP cells, which are susceptible to androgens, and VCaP and 22Rv1 models, which are castration-resistant, were used to validate these effects. The consistent *α*-mangostin effect in these models suggests an androgen-independent mechanism of activity. The study suggests that *α*-mangostin could function as a promising natural compound capable of overcoming challenging resistance mechanism in CRPC [111]. Mechanistic investigations into the cellular machinery responsible for AR degradation confirmed that the compound actively promotes this process, providing insight into how *α*-mangostin exerts its antiandrogenic effects [112].

A further, in-depth examination indicates that *α*-mangostin’s mechanism of action is intricate and distinct, markedly differing from conventional AR inhibitors. Research indicates that AR degradation is an active proteolytic process rather than a consequence of transcriptional repression. MG132 treatment reinstated AR expression, corroborating the function of proteasomal degradation. The endoplasmic reticulum chaperone protein BiP (Binding immunoglobulin protein), also referred to as GRP7, was identified as a major mediator [113]. Nauman et al. (2023) [92] discovered that *α*-mangostin selectively interacts with AR and BiP, indicating ubiquitination and subsequent proteasomal degradation of the complex. These findings have significant therapeutic ramifications as the active elimination of AR-V7 circumvents clinical resistance mechanisms associated with second-generation endocrine agents such as enzalutamide and abiraterone [112]. This transforms AR’s job from staying alive to getting rid of targets. The target degradation route is effective and has been tested in complicated preclinical conditions. In vivo xenograft studies showed a significant reduction in tumor growth with *α*-mangostin treatment, confirming the therapeutic significance and translational potential of this compound [114]. In general, *α*-mangostin is a protein-degrading agent capable of targeting AR and potentially overcoming therapeutic resistance in advanced prostate cancer [115]. Another effective strategy is to eliminate or suppress the hormone that activate the androgen receptor.

### 4.4. Modulation of the AR Pathway via Alternative Targets

This technique shows how natural chemicals can manipulate the cell’s metabolic ecology to indirectly inhibit AR signaling [116]. These chemicals target pathways and biological components beyond the nuclear AR protein to disrupt androgen-dependent cancer growth in novel ways [117]. To validate that a natural compound specifically targets the AR signaling pathway, it is essential to demonstrate differential activity between AR-positive (e.g., LNCaP, 22Rv1, VCaP) and AR-negative (e.g., PC-3, DU-145) prostate cancer models. Without such evidence, observed antiproliferative effects may stem from non-specific cytotoxicity rather than hormonal modulation.

For instance, neoisoliquiritin (NEO) exemplifies high AR-centric specificity; it significantly inhibits AR-dependent LNCaP proliferation while exhibiting minimal cytotoxicity in AR-independent PC-3 cells. Conversely, some studies included in our initial analysis, such as those involving tea catechins (EGCG) in the AR-negative DU-145 cell line, suggest a dualistic nature. While in silico docking reveals a high affinity for the AR ligand-binding domain (LBD), the observed in vitro lethality in AR-negative models indicates that these catechins likely exert significant AR-independent antiproliferative effects. This distinction is critical for clinical translation, as compounds with purely AR-dependent mechanisms are less likely to produce systemic off-target toxicities.

#### 4.4.1. Inhibition of the 5-α-Reductase Enzyme

The 5-*α*-reductase enzyme converts testosterone into DHT, acting as a key “gatekeeper” and amplifier in the androgen signaling cascade [118]. The most physiologically potent androgen is DHT, which binds and transactivates the AR better than testosterone [119]. Inhibition of this enzyme reduces AR pathway activation by disrupting DHT supply, the main AR ligand [120]. Recently, natural substances have been found to inhibit the enzymes involved in the synthesis of this potent hormone [121].

**Figure 5:**
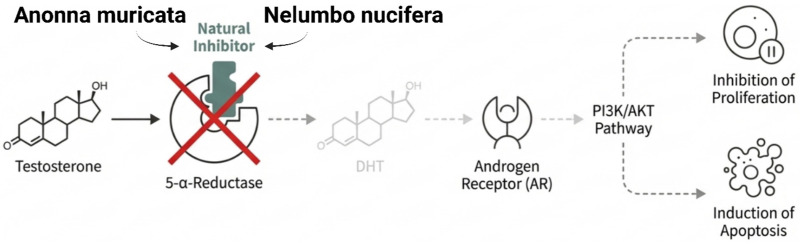
Inhibition of androgen biosynthesis by plant-derived alkaloids and acetogenins (created with BioRender.com).

Based on Figure 5, current therapeutic strategies primarily focus on depleting circulating androgens or direct receptor antagonism. Androgen deprivation therapy (ADT), involving surgical or chemical castration combined with antiandrogens like bicalutamide, remains a cornerstone of treatment. Advanced options, such as enzalutamide and abiraterone acetate, have improved survival outcomes by targeting receptor affinity and androgen biosynthesis, respectively. However, the eventual progression to CRPC highlights the limitations of existing standards and suggests a role for multitargeted natural scaffolds in future drug discovery.

The preventive research of *Annona muricata* and *Nelumbo nucifera* has become increasingly important in the search for natural antiandrogenic agents. Apeh et al. (2023) [100] conducted an in silico evaluation of bioactive compounds *Annona muricata* (soursop), providing strong support for their potential as prostate cancer therapeutic agent. This study involved computational screening of 263 phytochemical compounds from plants to determine their ability to inhibit 5-*α*-reductase 2 [100]. This enzyme converts testosterone into powerful dihydrotestosterone (DHT), the most potent endogenous ligand of the androgen receptor [122]. Many acetogenins were found as prime inhibitory candidates using molecular docking, MM-GBSA free energy calculations, and ADMET profiles. Similarly, alkaloids isolated from *Nelumbo nucifera* demonstrated notable capacity to suppress DHT synthesis, suggesting that lotus-derived compounds may also serve as effective natural inhibitors of hormonal activation pathways in prostate cancer.

Annopentocin-A and muricatetrocin-A had the highest expected binding affinity and structural stability, surpassing finasteride, the clinical inhibitor [100]. In vitro research on lotus seed alkaloids supports and experimentally verifies these in silico findings of high inhibitory capability [123]. Although the *Annona muricata* study provides computational evidence for inhibiting 5-*α*-reductase, the *Nelumbo nucifera* study shows that natural compounds can efficiently inhibit this target in prostate cancer cells [124]. These studies show a coordinated therapeutic strategy in which a variety of natural molecules identified by computational modeling and experimental validation converge on a critical enzymatic node to disrupt the androgen receptor’s most potent ligand. The lotus-seed-derived *bisbenzylisoquinoline* alkaloids neferine, liensinine, and isoliensinine have multimodal antiandrogenic effects. Their activity involves directly inhibiting the 5-*α*-reductase enzymatic pathway, thereby lowering testosterone conversion to DHT [94]. By suppressing DHT synthesis, these compounds indirectly inhibit AR activation and downstream oncogenic signaling [125]. However, not all natural agents act by blocking androgen production; some exert anticancer effects by activating specific cell-surface receptors to initiate tumor cell death [126].

#### 4.4.2. Activation of the Membrane Androgen Receptor (ZIP9)

**Figure 6:**
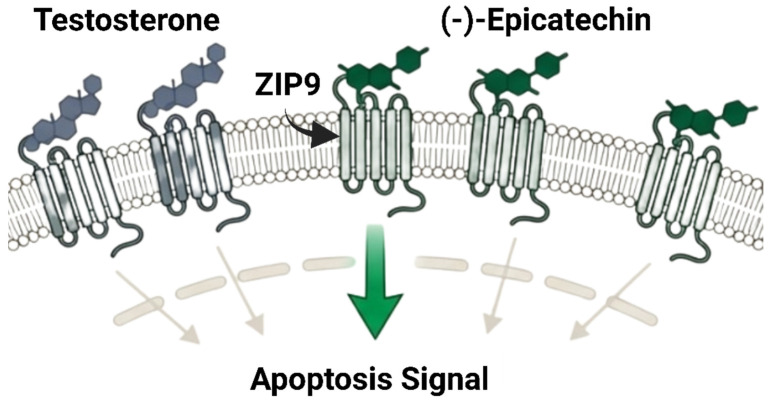
Non-classical androgen signaling modulation by (-)-epicatechin (created with BioRender.com).

In Figure 6, prostate tumors that have progressed beyond the traditional nuclear androgen receptor (nAR) may be vulnerable to alternative therapeutic strategies involving non-genomic, membrane-initiated androgen signaling. Thomas and Dong (2021) reported that (-)-epicatechin has a novel mechanism that challenges prostate cancer androgen signaling studies [31]. (-)-Epicatechin strongly binds to ZIP9 (SLC39A9), a membrane androgen receptor, rather than the traditional nAR [127]. (-)-Epicatechin binds ZIP9 with 75% of testosterone affinity, while (+)-catechin acts as an inhibitor [128]. By engaging ZIP9, (-)-epicatechin “hijacks” the androgen signaling cascade converting it into a proapoptotic response [31]. This activation triggers G-protein-mediated pathways, leading to caspases 3 and 9 activation and subsequent programmed cell death [127]. Interestingly, similar effects were observed in PC-3 prostate cancer cells, an nAR-negative cell line that resists traditional endocrine therapy [128]. This discovery shows that membrane-initiated testosterone signaling can induce death in prostate cancer cells without the nuclear AR target [129]. It introduces an innovative approach to treating advanced, therapy-resistant prostate malignancies beyond AR blocking [130]. Furthermore, some natural compounds demonstrate dual receptor modulation by influencing both androgen and estrogen signaling pathways.

#### 4.4.3. Dual Modulation of Androgen and Estrogen Receptors

The study by Khan et al. demonstrated that the furanoflavonoid karanjin may regulate the complex hormonal balance involved in prostate cancer progression through many complementary mechanisms [99]. In silico analyses revealed that karanjin function as a dual receptor modulator, with significant binding affinity for both the androgen receptor (AR) and estrogen receptor beta (ERβ) [131]. This dual mechanism indicates a broader therapeutic strategy, wherein a single medication can suppress the proproliferative AR pathway while simultaneously activating the antiproliferative ERβ pathway [132].

**Figure 7:**
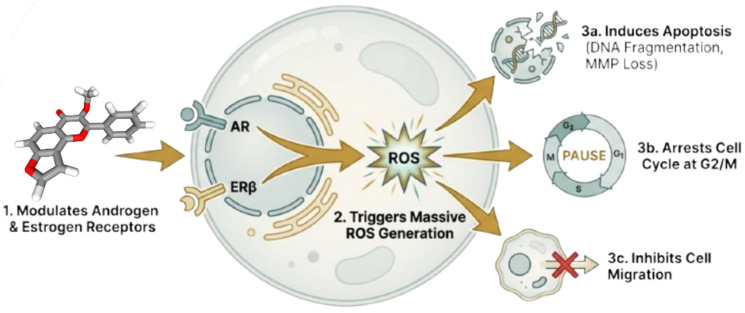
Modulation of dual receptors and the cytotoxic mechanism of Karanjin (created with BioRender.com).

In the Figure 7, Khan et al. further validated karanjin’s hormonal regulation and anticancer actions in the AR-negative PC-3 cell line [99]. The compound induces reactive oxygen species (ROS), leading to a G2/M cell cycle arrest and apoptosis. This mechanism significantly reduces the viability of cancer cells, similar to the actions of curcumin [133], quercetin [134], asperlin [135], and baicalein [136] on other cancer targets. Auriculasin [137] also targets the accumulation of reactive oxygen species (ROS), selectively eliminating damaged cells while sparing normal cells. This mechanism may contribute to its potential suppression of prostate cancer. These findings highlight karanjin as a multifunctional natural chemical that can directly induce ROS, disrupt the cell cycle, and affect the tumor microenvironment’s hormonal balance [138,139,140]. Its efficacy in androgen-independent prostate cancer animals suggests that it could cure advanced and treatment-resistant disease [141]. Nevertheless, despite these promising results, it is important to acknowledge the limitations of current studies and the uncertainty surrounding the translation of such compounds to human clinical application.

### 4.5. Limitations

This review highlights basic and preclinical data, as none of the investigated natural compounds have advanced to human clinical trials; therefore, their efficacy and safety in patients remained unestablished. This is one of the main limitations in the current studies. The 15 included studies used different cell lines such as LNCaP, 22Rv1, and PC-3 and multiple in silico studies resulting in findings that are often computationally predicted rather than biologically confirmed. Furthermore, many of these compounds cannot yet be quantified or standardized in a practical therapeutic context. Pharmacokinetic data for promising in vivo validated agents, such as *α*-mangostin and manzamine-A, remain sparse, particularly oral bioavailability, solubility, metabolism, and toxicity profiles. Consequently, a substantial translational gap persists, and future research should prioritize addressing these weaknesses through systematic pharmacokinetic characterization, standardized dosing, and eventual progression toward clinical evaluation.

### 4.6. Future and Prospect

This systematic review highlights the potential of natural compounds as promising AR modulators for prostatic cancer therapy. We identified a diverse range of bioactive molecules capable of altering AR signaling, such as *α*-mangostin, manzamine-A, and *α*-terthienyl, which have attracted considerable research interest due to their potent preclinical effects. However, substantial questions remain about their physiological mechanisms and pharmacokinetic properties, including absorption, solubility, metabolism, and toxicity. Overcoming these limitations will be essential to advancing these compounds toward clinical application. Future research that integrates these natural compounds with joint treatment medications such as enzalutamide may enhance efficacy and reduce resistance and broaden therapeutic options for advanced prostate cancer

Notably, *α*-mangostin demonstrates the ability to degrade both full-length AR and the therapy-resistant AR-V7 isoform. Manzamine-A inhibits AR synthesis by disrupting the regulatory transcription factor E2F8, while (-)-epicatechin, act through the non-classical ZIP9 membrane receptor, illustrating the mechanistic versatility of natural products. While natural compounds such as α-mangostin, manzamine A, and α-terthienyl have demonstrated significant potential in preclinical models, substantial questions regarding their pharmacokinetic properties remain. Therefore, these molecules should be considered promising lead candidates rather than immediate clinical solutions. Future research should prioritize improving bioavailability through optimized formulation strategies and investigating combinatorial therapies with established agents like enzalutamide.

## 5. Conclusions

This comprehensive assessment of 15 studies demonstrates that natural product derived compounds are versatile modulators of AR signaling, capable of exerting their effects through multiple mechanistic pathways. The study demonstrates that these bioactive compounds can directly suppress AR expression, activity, and nuclear translocation, while also engaging alternative and clinically relevant mechanisms that reshape therapeutic possibilities. Identifying compounds capable of overcoming resistance is a critical priority in prostate cancer treatment. In castration-resistant prostate cancer (CRPC), for example, *α*-Mangostin promotes proteasomal degradation of both full-length AR and the persistent AR-V7 splice variant, whereas manzamine-A inhibits E2F8 to prevent receptor synthesis. These represent novel approaches to circumventing endocrine resistance. Unconventional paradigms, such as the reutilization of (-)epicatechin in androgen signaling to trigger apoptosis via the ZIP9 non-classical membrane receptor, highlight the dynamic ability of natural compounds to reprogram cellular signaling for therapeutic benefits.

These findings provide a strong foundation for future pharmaceuticals development. Compounds such as *α*-mangostin, manzamine A, and α-terthienyl have emerged as potential candidates for clinical trials. Future efforts should focus on enhancing bioavailability by optimizing pharmacokinetics and formulations strategies. Investigating combinatorial therapies with established agents like enzalutamide may also boost efficacy and delay resistance. New AR signaling pathway molecular targets such as E2f8 and ZIP9 require comprehensive assessment as potential therapeutic entry points. Collectively, the diverse chemical scaffolds and unique modes of action identified in this review provide a scientific foundation for the development of next-generation prostate cancer therapeutics. However, human clinical trials are strictly necessary to translate these pre-clinical observations into safe and effective medical interventions.

## Figures and Tables

**Table 1:** Summary of bioactive natural compounds targeting androgen receptor signaling in prostate cancer.

No	Bioactive Compounds	Study Model	Key Findings	Key Mechanisms	Reference
1	*α*-mangostin	In vitro (LNCaP, 22Rv1, VCaP); in vivo (Xenograft).	*IC*_50_ (viability): ~10 µm; tumor reduction: >50% at 50 mg/kg.	Induces degradation of AR and AR-V7 via the BiP-mediated ubiquitin-proteasome pathway.	Nauman et al., 2023 [92]
2	*α*-terthienyl	In vitro (LNCaP, 22Rv1); in vivo (Xenograft).	*IC*_50_ (viability): ~2.5 µm; tumor reduction: significant suppression.	Inhibits nuclear translocation and suppresses the expression of AR protein and its target genes.	Gan et al., 2022 [93]
3	Neoisoliquiritin (NEO)	In vitro (LNCaP); in vivo (Xenograft).	*IC*_50_ (viability): ~15.8 µm; tumor reduction: significant suppression.	Inhibits the expression, nuclear translocation, and transcriptional activity of AR.	Chen et al., 2021 [26]
4	Neferine	In vitro (LNCaP, DU-145, PC-3).	*IC*_50_ (viability): ~12.5 µm.	Downregulates the expression of AR protein and the 5-*α*-reductase enzyme via PI3K/AKT inactivation.	Liu et al., 2021 [94]
5	*Vitis vinifera* (cis-piceid)	In silico (PDB ID: 2AXA)	Docking score: −10.3 kcal/mol.	In silico: Demonstrates high binding affinity to the AR ligand-binding domain.	Olubode et al., 2022 [95]
6	Manzamine A	In vitro (LNCaP, 22Rv1); in vivo (Xenograft).	*IC*_50_ (viability): ~1 µm; tumor reduction: Effective growth inhibitor.	Suppresses AR synthesis (AR-FL and AR-V7) by inhibiting the upstream transcription factor, E2F8.	Karan et al., 2024 [96]
7	Alpinumisoflavone (AIF)	In vitro (LNCaP, C4-2).	*IC*_50_ (viability): ~18.4–21.7 µm.	Suppresses the mRNA and protein expression of AR and its target gene, PSA.	Basavaraj et al. 2022 [27]
8	Tea Catechins (EGCG)	In vitro (DU-145); in silico (PDB ID: 5T8E).	*IC*_50_ (viability): ~43.8 µg/mL; docking score: −9.5 kcal/mol.	In silico: Demonstrates strong binding interaction with the AR protein.	Muriuki et al., 2024 [97]
9	Tangeretin	In vitro (C4-2, DU-145).	*IC*_50_ (viability): ~28.6 µm.	Downregulates the expression of AR protein and the related signaling pathway (AKT).	Zhang et al., 2022 [30]
10	Hispidin	In vitro (LNCaP, C4-2).	*IC*_50_ (viability): ~14.9–19.3 µm.	Reduces the expression level of AR protein in prostate cancer cells.	Chan et al., 2024 [74]
11	Baicalein	In vitro (LNCaP).	*IC*_50_ (viability): ~18.4 µm.	Decreases the transcriptional activity of the AR gene promoter.	Jiang et al., 2023 [29]
12	Lupeol	In vitro (LNCaP).	*IC*_50_ (viability): ~50.6 µm.	Decreases the gene expression level (mRNA) of AR.	Nezami Majd et al., 2024 [98]
13	(-)-Epicatechin	In vitro (PC-3 ZIP9).	Relative binding affinity (to ZIP9): 75% vs. testosterone.	Acts as an agonist at the membrane androgen receptor ZIP9, inducing apoptosis.	Thomas & Dong, 2021 [31]
14	Karanjin	In vitro (PC-3); in silico (PDB ID: AR 2Q7I, ERβ 1QKM).	*IC*_50_ (viability): ~34.8; docking score: −8.9 kcal/mol.	Demonstrates binding affinity for both AR and ERβ, modulating both.	Khan et al., 2025 [99]
15	*Annona muricata* (Annopentocin A)	In silico (PDB ID: 7BW1).	Docking score: −11.1 kcal/mol.	Inhibits the 5-α-reductase 2 enzyme, reducing the production of the potent AR ligand (DHT).	Apeh et al., 2023 [100]

## Data Availability

No new data were created or analyzed in this study. Data sharing is not applicable to this article as all analyzed data are included within the manuscript.

## References

[1] Zi H., Liu M.-Y., Luo L.-S., Huang Q., Luo P.-C., Luan H.-H., Huang J., Wang D.-Q., Wang Y.-B., Zhang Y.-Y. (2024). Global burden of benign prostatic hyperplasia, urinary tract infections, urolithiasis, bladder cancer, kidney cancer, and prostate cancer from 1990 to 2021. Mil. Med. Res..

[2] Sung H., Ferlay J., Siegel R.L., Laversanne M., Soerjomataram I., Jemal A., Bray F. (2021). Global Cancer Statistics 2020: GLOBOCAN Estimates of Incidence and Mortality Worldwide for 36 Cancers in 185 Countries. CA Cancer J. Clin..

[3] Ismuha R.R., Ritawidya R., Daruwati I., Muchtaridi M. (2024). Future Prospect of Low-Molecular-Weight Prostate-Specific Membrane Antigen Radioisotopes Labeled as Theranostic Agents for Metastatic Castration-Resistant Prostate Cancer. Molecules.

[4] Formaggio N., Rubin M.A., Theurillat J.-P. (2021). Loss and revival of androgen receptor signaling in advanced prostate cancer. Oncogene.

[5] Johnson M.J., Wasmuth E.V. (2024). Structural perspectives on the androgen receptor, the elusive shape-shifter. Steroids.

[6] Banerjee P.P., Srivastava S. (2024). Androgen Receptor Signaling: A Central and Evolving Theme in Prostate Cancer Treatment. Handbook of Oncobiology: From Basic to Clinical Sciences.

[7] Shiota M., Akamatsu S., Tsukahara S., Nagakawa S., Matsumoto T., Eto M. (2022). Androgen receptor mutations for precision medicine in prostate cancer. Endocr.-Relat. Cancer.

[8] Mateo J., Steuten L., Aftimos P., André F., Davies M., Garralda E., Geissler J., Husereau D., Martinez-Lopez I., Normanno N. (2022). Delivering precision oncology to patients with cancer. Nat. Med..

[9] Mohler M.L., Sikdar A., Ponnusamy S., Hwang D.-J., He Y., Miller D.D., Narayanan R. (2021). An Overview of Next-Generation Androgen Receptor-Targeted Therapeutics in Development for the Treatment of Prostate Cancer. Int. J. Mol. Sci..

[10] Banyal A., Tiwari S., Sharma A., Chanana I., Patel S.K.S., Kulshrestha S., Kumar P. (2023). Vinca alkaloids as a potential cancer therapeutics: Recent update and future challenges. 3 Biotech.

[11] Melfi F., Carradori S., Mencarelli N., Campestre C., Gallorini M., Di Giacomo S., Di Sotto A. (2023). Natural products as a source of new anticancer chemotypes. Expert Opin. Ther. Pat..

[12] Sun R., Guo F., Zhang Y., Shao H., Yang X., Wang C., Yang C. (2025). Biological activity of secondary metabolites of actinomycetes and their potential sources as antineoplastic drugs: A review. Front. Microbiol..

[13] Nižnanský Ľ., Osinová D., Kuruc R., Hengerics Szabó A., Szórádová A., Masár M., Nižnanská Ž. (2022). Natural Taxanes: From Plant Composition to Human Pharmacology and Toxicity. Int. J. Mol. Sci..

[14] Mali S.B. (2023). Cancer treatment: Role of natural products. Time to have a serious rethink. Oral Oncol. Rep..

[15] Nasim N., Sandeep I.S., Mohanty S. (2022). Plant-derived natural products for drug discovery: Current approaches and prospects. Nucleus.

[16] Bizzarri F.P., Campetella M., Ragonese M., Scarciglia E., Russo P., Marino F., Filomena G.B., Gavi F., Rossi F., D’Amico L. (2024). The role of alternative medicine and complimentary therapies in urologic disease: New horizons. Urol. J..

[17] Kristoffersen A.E., Nilsen J.V., Stub T., Nordberg J.H., Wider B., Mora D., Nakandi K., Bjelland M. (2022). Use of Complementary and Alternative Medicine in the context of cancer; prevalence, reasons for use, disclosure, information received, risks and benefits reported by people with cancer in Norway. BMC Complement. Med. Ther..

[18] Majrashi T.A., Alshehri S.A., Alsayari A., Muhsinah A.B., Alrouji M., Alshahrani A.M., Shamsi A., Atiya A. (2023). Insight into the Biological Roles and Mechanisms of Phytochemicals in Different Types of Cancer: Targeting Cancer Therapeutics. Nutrients.

[19] Tiwari S.S., Honmane S.M., Sarda R.R., Gattani S.G., Mahaparale P.R., Hoque M.E., Das Talukdar A., Patra J.K., Das G., Nath D. (2024). Economic Benefits of Natural Products in Modern Drug Discovery in the Twenty-First Century. Traditional Resources and Tools for Modern Drug Discovery: Ethnomedicine and Pharmacology.

[20] Muchtaridi M., Wijaya C.A. (2017). Anticancer potential of α-mangostin. Asian J. Pharm. Clin. Res..

[21] Herdiana Y., Wathoni N., Shamsuddin S., Muchtaridi M. (2021). α-Mangostin Nanoparticles Cytotoxicity and Cell Death Modalities in Breast Cancer Cell Lines. Molecules.

[22] Dewi C., Fristiohady A., Amalia R., Ikram N.K.K., Ibrahim S., Muchtaridi M. (2022). Signaling Pathways and Natural Compounds in Triple-Negative Breast Cancer Cell Line. Molecules.

[23] Tufail M., Jiang C.-H., Li N. (2024). Altered metabolism in cancer: Insights into energy pathways and therapeutic targets. Mol. Cancer.

[24] Abbas M., Saeed F., Anjum F.M., Afzaal M., Tufail T., Bashir M.S., Ishtiaq A., Hussain S., Suleria H.A.R. (2017). Natural polyphenols: An overview. Int. J. Food Prop..

[25] Bhosale P.B., Ha S.E., Vetrivel P., Kim H.H., Kim S.M., Kim G.S. (2020). Functions of polyphenols and its anticancer properties in biomedical research: A narrative review. Transl. Cancer Res..

[26] Chen C., Shao R., Li B., Zhai Y., Wang T., Li X., Miao L., Huang J., Liu R., Liu E. (2021). Neoisoliquiritin exerts tumor suppressive effects on prostate cancer by repressing androgen receptor activity. Phytomedicine.

[27] Basavaraj P., Ruangsai P., Hsieh P.-F., Jiang W.-P., Bau D.-T., Huang G.-J., Huang W.-C. (2022). Alpinumisoflavone Exhibits the Therapeutic Effect on Prostate Cancer Cells by Repressing AR and Co-Targeting FASN- and HMGCR-Mediated Lipid and Cholesterol Biosynthesis. Life.

[28] Huang S.-Y., Chang S.-F., Chau S.-F., Chiu S.-C. (2019). The Protective Effect of Hispidin against Hydrogen Peroxide-Induced Oxidative Stress in ARPE-19 Cells via Nrf2 Signaling Pathway. Biomolecules.

[29] Jiang M., Poudel S., Song K. (2023). Androgen receptor and hyaluronan-mediated motility receptor as new molecular targets of baicalein: New molecular mechanisms for its anticancer properties. Arch. Pharmacal Res..

[30] Zhang N., Wu W., Huang Y., An L., He Z., Chang Z., He Z., Lai Y. (2022). Citrus Flavone Tangeretin Inhibits CRPC Cell Proliferation by Regulating Cx26, AKT, and AR Signaling. Evid.-Based Complement. Altern. Med..

[31] Thomas P., Dong J. (2021). (-)-Epicatechin acts as a potent agonist of the membrane androgen receptor, ZIP9 (SLC39A9), to promote apoptosis of breast and prostate cancer cells. J. Steroid Biochem. Mol. Biol..

[32] Majeed Malik I., Hussain Bhat A., Majeed D., Nabi N. (2025). Environmental Factors Influencing Phytochemical Production for Enhanced Phytochemical Defense. Phytochemical Arsenal: Understanding Plant Defense Mechanisms Against Nematodes.

[33] Rudzińska A., Juchaniuk P., Oberda J., Wiśniewska J., Wojdan W., Szklener K., Mańdziuk S. (2023). Phytochemicals in Cancer Treatment and Cancer Prevention—Review on Epidemiological Data and Clinical Trials. Nutrients.

[34] Ahmed M.B., Islam S.U., Alghamdi A.A.A., Kamran M., Ahsan H., Lee Y.S. (2022). Phytochemicals as Chemo-Preventive Agents and Signaling Molecule Modulators: Current Role in Cancer Therapeutics and Inflammation. Int. J. Mol. Sci..

[35] Jang J.-H., Lee T.-J. (2023). Mechanisms of Phytochemicals in Anti-Inflammatory and Anti-Cancer. Int. J. Mol. Sci..

[36] Oyenihi O.R., Oyenihi A.B., Erhabor J.O., Matsabisa M.G., Oguntibeju O.O. (2021). Unravelling the Anticancer Mechanisms of Traditional Herbal Medicines with Metabolomics. Molecules.

[37] van Weverwijk A., de Visser K.E. (2023). Mechanisms driving the immunoregulatory function of cancer cells. Nat. Rev. Cancer.

[38] Efferth T., Koch E. (2011). Complex Interactions between Phytochemicals. The Multi-Target Therapeutic Concept of Phytotherapy. Curr. Drug Targets.

[39] Fu X., Zhang Z., Chen R., A J., An N., Tian X., Dong J.-T. (2025). ZFHX3 is integral to androgen/AR signaling involving protein association with AR in prostate cancer cells. Sci. Rep..

[40] He Y., Xu W., Xiao Y.-T., Huang H., Gu D., Ren S. (2022). Targeting signaling pathways in prostate cancer: Mechanisms and clinical trials. Signal Transduct. Target. Ther..

[41] Jacob A., Raj R., Allison D.B., Myint Z.W. (2021). Androgen Receptor Signaling in Prostate Cancer and Therapeutic Strategies. Cancers.

[42] Labaf M., Li M., Ting L., Karno B., Zhang S., Gao S., Patalano S., Macoska J.A., Zarringhalam K., Han D. (2022). Increased AR expression in castration-resistant prostate cancer rapidly induces AR signaling reprogramming with the collaboration of EZH2. Front. Oncol..

[43] Rana M., Dong J., Robertson M.J., Basil P., Coarfa C., Weigel N.L. (2021). Androgen receptor and its splice variant, AR-V7, differentially induce mRNA splicing in prostate cancer cells. Sci. Rep..

[44] Gómez Rivas J., Fernandez L., Abad-Lopez P., Moreno-Sierra J. (2023). Androgen deprivation therapy in localized prostate cancer. Current status and future trends. Actas Uról. Esp. (Engl. Ed.).

[45] Harris A.E., Metzler V.M., Lothion-Roy J., Varun D., Woodcock C.L., Haigh D.B., Endeley C., Haque M., Toss M.S., Alsaleem M. (2022). Exploring anti-androgen therapies in hormone dependent prostate cancer and new therapeutic routes for castration resistant prostate cancer. Front. Endocrinol..

[46] Facchini G., D’Arienzo A., Nicastro A., Flauto F., Izzo M., Montella L., Riccardo F., Fusco G.M., Trama F., Lauro G.D. (2025). Apalutamide Monotherapy in Metastatic Hormone-Sensitive Prostate Cancer: A Viable Alternative to First-Generation Anti-Androgen Agents to Avoid the Flare Phenomenon and an Effective Treatment for Achieving Early PSA Response. Cancers.

[47] Hadzi-Djokic J., Kocic G., Hadzi-Djokic J., Simic T. (2024). Hormone Therapy for Advanced Prostate Cancer. Prostate Cancer: Advancements in the Pathogenesis, Diagnosis and Personalized Therapy.

[48] Yu E.-m., Aragon-Ching J.B. (2022). Advances with androgen deprivation therapy for prostate cancer. Expert Opin. Pharmacother..

[49] Huang J., Lin B., Li B. (2022). Anti-Androgen Receptor Therapies in Prostate Cancer: A Brief Update and Perspective. Front. Oncol..

[50] Kuzminac I.Z., Nikolić A.R., Savić M.P., Ajduković J.J. (2024). Abiraterone and Galeterone, Powerful Tools Against Prostate Cancer: Present and Perspective. Pharmaceutics.

[51] Sharma K. (2024). Identification and Targeting of Novel Mechanisms for Treatment of Castration Resistant Prostate Cancer (CRPC). Ph.D. Thesis.

[52] Snaterse G., Taylor A.E., Moll J.M., O’Neil D.M., Teubel W.J., van Weerden W.M., Arlt W., Hofland J. (2024). Prostate cancer androgen biosynthesis relies solely on CYP17A1 downstream metabolites. J. Steroid Biochem. Mol. Biol..

[53] Wang B.-R., Chen Y.-A., Kao W.-H., Lai C.-H., Lin H., Hsieh J.-T. (2022). Developing New Treatment Options for Castration-Resistant Prostate Cancer and Recurrent Disease. Biomedicines.

[54] Crowley F., Sterpi M., Buckley C., Margetich L., Handa S., Dovey Z. (2021). A Review of the Pathophysiological Mechanisms Underlying Castration-resistant Prostate Cancer. Res. Rep. Urol..

[55] Pozas J., Álvarez Rodríguez S., Fernández V.A., Burgos J., Santoni M., Manneh Kopp R., Molina-Cerrillo J., Alonso-Gordoa T. (2022). Androgen Receptor Signaling Inhibition in Advanced Castration Resistance Prostate Cancer: What Is Expected for the Near Future?. Cancers.

[56] Quistini A., Chierigo F., Fallara G., Depalma M., Tozzi M., Maggi M., Jannello L.M.I., Pellegrino F., Mantica G., Terracciano D. (2025). Androgen Receptor Signalling in Prostate Cancer: Mechanisms of Resistance to Endocrine Therapies. Res. Rep. Urol..

[57] Tulk J., Rash J.A., Thoms J., Wassersug R., Gonzalez B., Garland S.N. (2023). Androgen deprivation therapy and radiation for prostate cancer—Cognitive impairment, sleep, symptom burden: A prospective study. BMJ Support. Palliat. Care.

[58] Ussing A., Mikkelsen M.-L.K., Villumsen B.R., Wejlgaard J., Bistrup P.E., Birkefoss K., Bandholm T. (2022). Supervised exercise therapy compared with no exercise therapy to reverse debilitating effects of androgen deprivation therapy in patients with prostate cancer: A systematic review and meta-analysis. Prostate Cancer Prostatic Dis..

[59] Naithani U., Guleria V. (2024). Integrative computational approaches for discovery and evaluation of lead compound for drug design. Front. Drug Discov..

[60] Gupta R., Srivastava D., Sahu M., Tiwari S., Ambasta R.K., Kumar P. (2021). Artificial intelligence to deep learning: Machine intelligence approach for drug discovery. Mol. Divers..

[61] Biju T.S., Priya V.V., Francis A.P. (2023). Role of three-dimensional cell culture in therapeutics and diagnostics: An updated review. Drug Deliv. Transl. Res..

[62] Niazi S.K. (2023). A Critical Analysis of the FDA’s Omics-Driven Pharmacodynamic Biomarkers to Establish Biosimilarity. Pharmaceuticals.

[63] Kim C., Kim B. (2018). Anti-Cancer Natural Products and Their Bioactive Compounds Inducing ER Stress-Mediated Apoptosis: A Review. Nutrients.

[64] Kallifatidis G., Hoy J.J., Lokeshwar B.L. (2016). Bioactive natural products for chemoprevention and treatment of castration-resistant prostate cancer. Semin. Cancer Biol..

[65] Makino T., Izumi K., Mizokami A. (2021). Undesirable Status of Prostate Cancer Cells after Intensive Inhibition of AR Signaling: Post-AR Era of CRPC Treatment. Biomedicines.

[66] Zhang Y.-L., Chen G.-L., Liu Y., Zhuang X.-C., Guo M.-Q. (2021). Stimulation of ROS Generation by Extract of Warburgia ugandensis Leading to G0/G1 Cell Cycle Arrest and Antiproliferation in A549 Cells. Antioxidants.

[67] Siciliano T., Simons I.H., Beier A.-M.K., Ebersbach C., Aksoy C., Seed R.I., Stope M.B., Thomas C., Erb H.H.H. (2021). A Systematic Comparison of Antiandrogens Identifies Androgen Receptor Protein Stability as an Indicator for Treatment Response. Life.

[68] Hao J., Ci X., Xue H., Wu R., Dong X., Choi S.Y.C., He H., Wang Y., Zhang F., Qu S. (2018). Patient-derived Hormone-naive Prostate Cancer Xenograft Models Reveal Growth Factor Receptor Bound Protein 10 as an Androgen Receptor-repressed Gene Driving the Development of Castration-resistant Prostate Cancer. Eur. Urol..

[69] Devlies W., Handle F., Devos G., Joniau S., Claessens F. (2021). Preclinical Models in Prostate Cancer: Resistance to AR Targeting Therapies in Prostate Cancer. Cancers.

[70] Beretta G.L., Zaffaroni N. (2019). Androgen Receptor-Directed Molecular Conjugates for Targeting Prostate Cancer. Front. Chem..

[71] Lee S., Hoang G.D., Kim D., Song H.S., Choi S., Lee D., Kang K.S. (2021). Efficacy of Alpinumisoflavone Isolated from Maclura tricuspidata Fruit in Tumor Necrosis Factor-α-Induced Damage of Human Dermal Fibroblasts. Antioxidants.

[72] Wang T., Jiang Y., Chu L., Wu T., You J. (2017). Alpinumisoflavone suppresses tumour growth and metastasis of clear-cell renal cell carcinoma. Am. J. Cancer Res..

[73] Ateba S.B., Mvondo M.A., Djiogue S., Zingué S., Krenn L., Njamen D. (2019). A Pharmacological Overview of Alpinumisoflavone, a Natural Prenylated Isoflavonoid. Front. Pharmacol..

[74] Chan K.C., Basavaraj P., Tsai J.C., Viehoever J., Hsieh B.Y., Li X.Y., Huang G.J., Huang W.C. (2024). Evaluating the Therapeutic Effect of Hispidin on Prostate Cancer Cells. Int. J. Mol. Sci..

[75] Oguić R., Mozetič V., Cini Tešar E., Fučkar Čupić D., Mustać E., Dorđević G. (2014). Matrix metalloproteinases 2 and 9 immunoexpression in prostate carcinoma at the positive margin of radical prostatectomy specimens. Pathol. Res. Int..

[76] Justulin L.A., Della-Coleta H.H.M., Taboga S.R., Felisbino S.L. (2010). Matrix metalloproteinase (MMP)-2 and MMP-9 activity and localization during ventral prostate atrophy and regrowth. Int. J. Androl..

[77] Li H., Qiu Z., Li F., Wang C. (2017). The relationship between MMP-2 and MMP-9 expression levels with breast cancer incidence and prognosis. Oncol. Lett..

[78] Shoari A., Ashja Ardalan A., Dimesa A.M., Coban M.A. (2024). Targeting Invasion: The Role of MMP-2 and MMP-9 Inhibition in Colorectal Cancer Therapy. Biomolecules.

[79] Młynarczyk G., Gudowska-Sawczuk M., Mroczko B., Bruczko-Goralewska M., Romanowicz L., Tokarzewicz A. (2023). Higher Content but No Specific Activity in Gelatinase B (MMP-9) Compared with Gelatinase A (MMP-2) in Human Renal Carcinoma. Cancers.

[80] Radisky E.S. (2024). Extracellular proteolysis in cancer: Proteases, substrates, and mechanisms in tumor progression and metastasis. J. Biol. Chem..

[81] Zhu Z., Cao Y., Liu L., Zhao Z., Yin H., Wang H. (2022). Atorvastatin regulates the migration and invasion of prostate cancer through the epithelial-mesenchymal transformation and matrix metalloproteinase pathways. Investig. Clin. Urol..

[82] Tiwari A., Mukherjee B., Hassan M.K., Pattanaik N., Jaiswal A.M., Dixit M. (2019). Reduced FRG1 expression promotes prostate cancer progression and affects prostate cancer cell migration and invasion. BMC Cancer.

[83] Liu J., Han X., Zhang T., Tian K., Li Z., Luo F. (2023). Reactive oxygen species (ROS) scavenging biomaterials for anti-inflammatory diseases: From mechanism to therapy. J. Hematol. Oncol..

[84] Chen J., Gou Z., Yang G., Zhou L., Kim A.N., Shi W., Zhou Y. (2025). Ferroptosis, a Distinct Form of Cell Death, and Research Progress on Its Modulators. Pharmaceuticals.

[85] Draker R., Sarcinella E., Cheung P. (2011). USP10 deubiquitylates the histone variant H2A.Z and both are required for androgen receptor-mediated gene activation. Nucleic Acids Res..

[86] Liu H., Dong Y., Gao Y., Du Z., Wang Y., Cheng P., Chen A., Huang H. (2016). The Fascinating Effects of Baicalein on Cancer: A Review. Int. J. Mol. Sci..

[87] Morshed A.K.M.H., Paul S., Hossain A., Basak T., Hossain M.S., Hasan M.M., Hasibuzzaman M.A., Rahaman T.I., Mia M.A.R., Shing P. (2023). Baicalein as Promising Anticancer Agent: A Comprehensive Analysis on Molecular Mechanisms and Therapeutic Perspectives. Cancers.

[88] Lai Y., Wu W., Liang X., Zhong F., An L., Chang Z., Cai C., He Z., Wu W. (2023). Connexin43 is associated with the progression of clear cell renal carcinoma and is regulated by tangeretin to sygergize with tyrosine kinase inhibitors. Transl. Oncol..

[89] Ashrafizadeh M., Ahmadi Z., Mohammadinejad R., Afshar E.G. (2020). Tangeretin: A mechanistic review of its pharmacological and therapeutic effects. J. Basic Clin. Physiol. Pharmacol..

[90] Alhamad D.W., Elgendy S.M., Al-Tel T.H., Omar H.A. (2021). Tangeretin as an adjuvant and chemotherapeutic sensitizer against various types of cancers: A comparative overview. J. Pharm. Pharmacol..

[91] de Mejia E.G., Ramirez-Mares M.V., Puangpraphant S. (2009). Bioactive components of tea: Cancer, inflammation and behavior. Brain Behav. Immun..

[92] Nauman M.C., Won J.H., Petiwala S.M., Vemu B., Lee H., Sverdlov M., Johnson J.J. (2023). alpha-Mangostin Promotes In Vitro and In Vivo Degradation of Androgen Receptor and AR-V7 Splice Variant in Prostate Cancer Cells. Cancers.

[93] Gan X., Huang H., Wen J., Liu K., Yang Y., Li X., Fang G., Liu Y., Wang X. (2022). alpha-Terthienyl induces prostate cancer cell death through inhibiting androgen receptor expression. Biomed. Pharmacother..

[94] Liu C.M., Wu Z., Pan B., An L., Zhu C., Zhou J., Jiang Y. (2021). The antiandrogenic effect of neferine, liensinine, and isoliensinine by inhibiting 5-alpha-reductase and androgen receptor expression via PI3K/AKT signaling pathway in prostate cancer. Pharmazie.

[95] Olubode S.O., Bankole M.O., Akinnusi P.A., Adanlawo O.S., Ojubola K.I., Nwankwo D.O., Edjebah O.E., Adebesin A.O., Ayodele A.O. (2022). Molecular Modeling Studies of Natural Inhibitors of Androgen Signaling in Prostate Cancer. Cancer Inform..

[96] Karan D., Dubey S., Gunewardena S., Iczkowski K.A., Singh M., Liu P., Poletti A., Choo Y.M., Chen H.Z., Hamann M.T. (2024). Manzamine A reduces androgen receptor transcription and synthesis by blocking E2F8-DNA interactions and effectively inhibits prostate tumor growth in mice. Mol. Oncol..

[97] Muriuki J., Uwanyagasani G., Maina E., Irungu B., Khamadi S., Lwembe R., Adan A., Barmasai S., Ndacyayisenga J. (2024). Assessing the antiproliferative properties of various teas against the DU-145 prostate cancer cell line: A combined in vitro and in silico investigation. Phytomed. Plus.

[98] Nezami Majd M., Alizadeh A., Zadeh Hashem E. (2024). Effects of Lupeol on Estrogen and Androgen Receptor-Positive Breast and Prostate Cancer Cells: Lupeol in ER+ and AR+ BC and PC cells. Arch. Breast Cancer.

[99] Khan H., Azad I., Arif Z., Nasibullah M., Khan M.F., Arshad M. (2025). Computational and biological insights for anti-cancer effects of Karanjin through AR/ER modulation, ROS generation and cell cycle arrest in prostate cancer. J. Tradit. Complement. Med..

[100] Apeh V.O., Adegboyega A.E., Chukwuma I.F., Ugwah-Oguejiofor C.J., Aja P.M., Ofeimun J.O., Ale B.A., Johnson G.I., Ebenyi L.N., Iwaloye O. (2023). An in silico study of bioactive compounds of Annona muricata in the design of ani-prostate cancer agent: MM/GBSA, pharmacophore modeling and ADMET parameters. Inform. Med. Unlocked.

[101] Asano M., Hitaka T., Imada T., Yamada M., Morimoto M., Shinohara H., Hara T., Yamaoka M., Santou T., Nakayama M. (2017). Synthesis and biological evaluation of novel selective androgen receptor modulators (SARMs). Part II: Optimization of 4-(pyrrolidin-1-yl)benzonitrile derivatives. Bioorganic Med. Chem. Lett..

[102] Hsieh T.-C., Wu J.M. (2009). Targeting CWR22R*v*1 Prostate Cancer Cell Proliferation and Gene Expression by Combinations of the Phytochemicals EGCG, Genistein and Quercetin. Anticancer Res..

[103] Zhang F., Biswas M., Massah S., Lee J., Lingadahalli S., Wong S., Wells C., Foo J., Khan N., Morin H. (2022). Dynamic phase separation of the androgen receptor and its coactivators key to regulate gene expression. Nucleic Acids Res..

[104] Pungsrinont T., Kallenbach J., Baniahmad A. (2021). Role of PI3K-AKT-mTOR Pathway as a Pro-Survival Signaling and Resistance-Mediating Mechanism to Therapy of Prostate Cancer. Int. J. Mol. Sci..

[105] Doghish A.S., Mageed S.S.A., Zaki M.B., Abd-Elmawla M.A., Sayed G.A., Hatawsh A., Aborehab N.M., Moussa R., Mohammed O.A., Abdel-Reheim M.A. (2025). Role of long non-coding RNAs and natural products in prostate cancer: Insights into key signaling pathways. Funct. Integr. Genom..

[106] Zhang T., Karsh L.I., Nissenblatt M.J., Canfield S.E. (2020). Androgen Receptor Splice Variant, AR-V7, as a Biomarker of Resistance to Androgen Axis-Targeted Therapies in Advanced Prostate Cancer. Clin. Genitourin. Cancer.

[107] Zheng Z., Li J., Liu Y., Shi Z., Xuan Z., Yang K., Xu C., Bai Y., Fu M., Xiao Q. (2022). The Crucial Role of AR-V7 in Enzalutamide-Resistance of Castration-Resistant Prostate Cancer. Cancers.

[108] Rahman M., Akter K., Ahmed K.R., Fahim M.M., Aktary N., Park M.N., Shin S.-W., Kim B. (2024). Synergistic Strategies for Castration-Resistant Prostate Cancer: Targeting AR-V7, Exploring Natural Compounds, and Optimizing FDA-Approved Therapies. Cancers.

[109] Dhital B., Santasusagna S., Kirthika P., Xu M., Li P., Carceles-Cordon M., Soni R.K., Li Z., Hendrickson R.C., Schiewer M.J. (2023). Harnessing transcriptionally driven chromosomal instability adaptation to target therapy-refractory lethal prostate cancer. Cell Rep. Med..

[110] Mishan M.A., Choo Y.-M., Winkler J., Hamann M.T., Karan D. (2025). Manzamine A: A promising marine-derived cancer therapeutic for multi-targeted interactions with E2F8, SIX1, AR, GSK-3β, and V-ATPase-A systematic review. Eur. J. Pharmacol..

[111] Li M., Wang L., Lin D., Liu Z., Wang H., Yang Y., Sun C., Ye J., Liu Y. (2024). Advanced Bioinspired Multifunctional Platforms Focusing on Gut Microbiota Regulation. ACS Nano.

[112] Khamthong N., Hutadilok-Towatana N. (2017). Phytoconstituents and Biological Activities of *Garcinia Dulcis* (Clusiaceae): A Review. Nat. Prod. Commun..

[113] Erzurumlu Y., Catakli D., Sezer S. (2023). Cannabidiol Negatively Regulates Androgenic Signal in Prostate Cancer Cells and Fine-Tunes the Tumorigenesis by Modulating Endoplasmic Reticulum-Associated Degradation, Unfolded Protein Response, and Autophagy. Rev. Bras. Farmacogn..

[114] Shibata M.-A., Iinuma M., Morimoto J., Kurose H., Akamatsu K., Okuno Y., Akao Y., Otsuki Y. (2011). α-Mangostin extracted from the pericarp of the mangosteen (*Garcinia mangostana* Linn) reduces tumor growth and lymph node metastasis in an immunocompetent xenograft model of metastatic mammary cancer carrying a p53 mutation. BMC Med..

[115] Su J.-Y., Li W.-H., Li Y.-M. (2022). New opportunities for immunomodulation of the tumour microenvironment using chemical tools. Chem. Soc. Rev..

[116] Sarkar F.H., Li Y. (2004). Cell signaling pathways altered by natural chemopreventive agents. Mutat. Res. Fundam. Mol. Mech. Mutagen..

[117] Foley C., Mitsiades N. (2016). Moving Beyond the Androgen Receptor (AR): Targeting AR-Interacting Proteins to Treat Prostate Cancer. Horm. Cancer.

[118] Knudsen K.E., Kelly W.K. (2011). Outsmarting androgen receptor: Creative approaches for targeting aberrant androgen signaling in advanced prostate cancer. Expert Rev. Endocrinol. Metab..

[119] Heinlein C.A., Chang C. (2002). The Roles of Androgen Receptors and Androgen-Binding Proteins in Nongenomic Androgen Actions. Mol. Endocrinol..

[120] Aggarwal S., Thareja S., Verma A., Bhardwaj T.R., Kumar M. (2010). An overview on 5α-reductase inhibitors. Steroids.

[121] Srivilai J., Minale G., Scholfield C.N., Ingkaninan K. (2019). Discovery of Natural Steroid 5 Alpha-Reductase Inhibitors. ASSAY Drug Dev. Technol..

[122] Azzouni F., Godoy A., Li Y., Mohler J. (2012). The 5 Alpha-Reductase Isozyme Family: A Review of Basic Biology and Their Role in Human Diseases. Adv. Urol..

[123] Hasannejad-Asl B., Pooresmaeil F., Azadi S., Najafi A., Esmaeili A., Bagheri-Mohammadi S., Kazemi B. (2025). Computational drug discovery of potential 5α-reductase phytochemical inhibitors and hair growth promotion using in silico techniques. Front. Bioinform..

[124] Liu C.-M., Shao Z., Chen X., Chen H., Su M., Zhang Z., Wu Z., Zhang P., An L., Jiang Y. (2023). Neferine attenuates development of testosterone-induced benign prostatic hyperplasia in mice by regulating androgen and TGF-β/Smad signaling pathways. Saudi Pharm. J..

[125] Audet-Walsh É., Yee T., Tam I.S., Giguère V. (2017). Inverse Regulation of DHT Synthesis Enzymes 5α-Reductase Types 1 and 2 by the Androgen Receptor in Prostate Cancer. Endocrinology.

[126] Özören N., El-Deiry W.S. (2003). Cell surface Death Receptor signaling in normal and cancer cells. Semin. Cancer Biol..

[127] Thomas P., Pang Y., Dong J. (2017). Membrane androgen receptor characteristics of human ZIP9 (SLC39A) zinc transporter in prostate cancer cells: Androgen-specific activation and involvement of an inhibitory G protein in zinc and MAP kinase signaling. Mol. Cell. Endocrinol..

[128] Thomas P., Converse A., Berg H.A. (2018). ZIP9, a novel membrane androgen receptor and zinc transporter protein. Gen. Comp. Endocrinol..

[129] Kampa M., Pelekanou V., Castanas E. (2008). Membrane-initiated steroid action in breast and prostate cancer. Steroids.

[130] Moreira-Silva F., Henrique R., Jerónimo C. (2022). From Therapy Resistance to Targeted Therapies in Prostate Cancer. Front. Oncol..

[131] Figueira M.I., Carvalho T.M.A., Macário-Monteiro J., Cardoso H.J., Correia S., Vaz C.V., Duarte A.P., Socorro S. (2024). The Pros and Cons of Estrogens in Prostate Cancer: An Update with a Focus on Phytoestrogens. Biomedicines.

[132] Mahmoud A.M. (2013). Androgen Receptor Mutations and Estrogen Receptor-β Modulate Genistein Effects on Prostate Cancer Cells. Ph.D. Thesis.

[133] Weir N.M., Selvendiran K., Kutala V.K., Tong L., Vishwanath S., Rajaram M., Tridandapani S., Anant S., Kuppusamy P. (2007). Curcumin induces G2/M arrest and apoptosis in cisplatin-resistant human ovarian cancer cells by modulating Akt and p38 MAPK. Cancer Biol. Ther..

[134] Tubtimsri S., Chuenbarn T., Manmuan S. (2025). Quercetin triggers cell apoptosis-associated ROS-mediated cell death and induces S and G2/M-phase cell cycle arrest in KON oral cancer cells. BMC Complement. Med. Ther..

[135] He L., Nan M.-H., Oh H.C., Kim Y.H., Jang J.H., Erikson R.L., Ahn J.S., Kim B.Y. (2011). Asperlin induces G2/M arrest through ROS generation and ATM pathway in human cervical carcinoma cells. Biochem. Biophys. Res. Commun..

[136] Chuang T.-C., Shao W.-S., Hsu S.-C., Lee S.-L., Kao M.-C., Wang V. (2023). Baicalein Induces G2/M Cell Cycle Arrest Associated with ROS Generation and CHK2 Activation in Highly Invasive Human Ovarian Cancer Cells. Molecules.

[137] Cho H.-D., Lee J.-H., Moon K.-D., Park K.-H., Lee M.-K., Seo K.-I. (2018). Auriculasin-induced ROS causes prostate cancer cell death via induction of apoptosis. Food Chem. Toxicol..

[138] Nakamura H., Takada K. (2021). Reactive oxygen species in cancer: Current findings and future directions. Cancer Sci..

[139] Zhang Z., Li M., Zhang X., Zhou F. (2024). Novel Strategies for Tumor Treatment: Harnessing ROS-Inducing Active Ingredients from Traditional Chinese Medicine Through Multifunctional Nanoformulations. Int. J. Nanomed..

[140] Ma N., Wang Y., Li X., Xu M., Tan D. (2025). Reactive oxygen species in cancer: Mechanistic insights and therapeutic innovations. Cell Stress Chaperones.

[141] Germain L., Lafront C., Paquette V., Neveu B., Paquette J.-S., Pouliot F., Audet-Walsh É. (2023). Preclinical models of prostate cancer—Modelling androgen dependency and castration resistance in vitro, ex vivo and in vivo. Nat. Rev. Urol..

